# The song that never ends: The effect of repeated exposure on the development of an earworm

**DOI:** 10.1177/17470218231152368

**Published:** 2023-02-04

**Authors:** Callula Killingly, Philippe Lacherez

**Affiliations:** 1 School of Psychology & Counselling, Faculty of Health, Queensland University of Technology (QUT), Brisbane, QLD, Australia; 2 School of Early Childhood and Inclusive Education, Faculty of Education, Queensland University of Technology (QUT), Brisbane, QLD, Australia

**Keywords:** Earworm, involuntary musical imagery, repeated exposure

## Abstract

An “earworm”—the experience of a catchy melody that repeats persistently in the mind—is a ubiquitous yet mysterious cognitive phenomenon. Previous research demonstrates that earworms for vocal music engage working memory resources, manifesting as “inner singing.” This study investigated whether this effect is moderated by prior exposure to music. In one experimental session, participants (*N* = 44) were presented with four novel song choruses. To manipulate exposure, each song was presented between one and four times, counterbalanced across participants. The following day, participants undertook a serial recall task during and following presentation of each song. In addition, they rated the music on familiarity, enjoyment, their desire to sing along, and perceived catchiness, both before and following the experiment. Increased exposure to novel songs on the first day tended to result in greater interference on task performance during and following their presentation on the second day, yet the effect varied depending on the song. Ratings of the desire to sing along and perceived familiarity increased significantly between the sessions for all songs. These findings are important in understanding the relative influence of familiarity and song-level characteristics on the development of an earworm.

Music “stuck” in the head—also known as an “earworm”—is a curious phenomenon characterised by the involuntary repetition of a musical excerpt in the mind (Beaman & Williams, 2010; Halpern & Bartlett, 2011; Hyman et al., 2013). For the most, these segments tend to be short, simple, and repetitive (Beaman & Williams, 2010; Hyman et al., 2013; Kellaris, 2001), typically elicited by vocal music (Halpern & Bartlett, 2011; Hyman et al., 2013), but also reported for instrumental music (Floridou et al., 2017; Liikkanen, 2011; Moeck et al., 2018). Earworms appear to be ubiquitous (Liikkanen et al., 2015), with large-scale research indicating that they are a regular experience for most individuals (Liikkanen, 2008).

Much of the research in this area employs participant self-report to corroborate the experience of an earworm (Beaman et al., 2015; Beaman & Williams, 2010; Hyman et al., 2013; Jakubowski et al., 2017; Liikkanen, 2012), which several studies have complemented with neural imaging techniques (Gabriel et al., 2016; King, 2006). One potential limitation of self-report in the study of involuntary mental phenomena is that individuals may lack insight into their mental processes or may not recall their experiences accurately in the case of retrospective questionnaires. Furthermore, informing participants that the research concerns “earworms” may in fact elicit the experience, thereby producing demand characteristics.

To overcome limitations associated with self-report, our previous research (Killingly et al., 2021) sought to establish a methodology for indirectly assessing the experience of an earworm. Given the connection between singing behaviours and the experience of an earworm (McCullough-Campbell & Margulis, 2015; Müllensiefen et al., 2014; Williamson & Jilka, 2013), we proposed that an earworm episode involves the internal “singing along” with a song, with or without conscious awareness of the observer. To test this theory, we employed an experimental working memory paradigm to investigate whether verbal working memory is engaged during an earworm episode. Two experiments demonstrated that subvocal articulatory processes, as measured on a serial recall task, are disrupted following the presentation of familiar, catchy songs that compel one to sing along, suggesting that these particular songs continued to be rehearsed in working memory even after their presentation. In each of the experiments, the extent of this interference was moderated by how much people wanted to sing along, providing support for the idea that this effect is driven by internal singing.

The finding that concurrently presented music interferes with working memory performance is well established in previous literature (Alley & Greene, 2008; Perham & Sykora, 2012; Pring & Walker, 1994; Salamé & Baddeley, 1989). Known as the *Irrelevant Sound Effect*, this disruption to serial recall was initially demonstrated with background speech yet not with stationary background noise (Colle & Welsh, 1976; Salamé & Baddeley, 1982, 1989), leading to the early supposition that the interference was due to the phonological similarity between the to-be-ignored sound and the to-be-remembered information. However, this view did not account for findings in which the effect is also produced by some non-speech materials, such as fluctuating tones and music (Jones & Macken, 1993; Salamé & Baddeley, 1989). Hence, rather than phonological resemblance between competing information driving interference, the most current account of this phenomenon explains performance deficits as arising from the extent of variation within the sequence of background sounds (“changing state” effect; see Jones et al., 1992) and the disruptive effect this has on subvocal articulatory planning processes (Jones et al., 2004).

Notably, the effect observed in Killingly et al. (2021), in which serial recall performance is disrupted *following* the presentation of an irrelevant sound, and *during*, was not previously observed by Macken et al. (1999) in a study specifically looking for proactive effects of irrelevant sound. However, the stimuli employed by Macken et al. were non-musical (speech), which potentially makes music “special” in this respect. Importantly, the development of an experimental working memory paradigm to measure earworm experiences has provided a cognitive index of a song stuck in the head that is independent of self-report, enabling further investigation of the factors that influence the development and maintenance of earworms.

A key question in the literature is whether there is something intrinsically “sticky” about music which tends to elicit earworms, or whether any song or instrumental piece might get stuck in the head given sufficient exposure (Byron & Fowles, 2015; Jakubowski et al., 2017). Studies involving computational musical feature analyses have demonstrated that various musical features seem to underlie the songs most commonly self-reported as earworms (e.g., duration of notes, contour of melody; Finkel et al., 2010; Jakubowski et al., 2017). Along the same lines, research using similar methodologies shows there are certain melodic features related to people’s desire to sing along with a song (Pawley & Mullensiefen, 2012), and how memorable a novel piece of music is (Müllensiefen & Halpern, 2014). Accordingly, it may be that an earworm episode is driven by characteristics intrinsic to a piece of music.

However, research also indicates the importance of being repeatedly exposed to music in the development of earworms (Byron & Fowles, 2015; Liikkanen, 2012). Adding further complexity, it seems probable that both catchiness of a song and people’s exposure to it tend to be mutually reinforcing. For instance, particularly catchy music may be given more airtime, resulting in increased exposure, which could explain the tendency of these musical excerpts to get stuck in the head. In addition, the mere process of repetition can exert an influence on the perception of catchiness (e.g., the effect of “mere exposure”; Zajonc, 1968), and so a song’s inherent “catchiness” becomes confounded with how often it is presented. Beyond the effects of the radio/television, people tend to listen and relisten to music that they enjoy in the home and on personal devices (Conrad et al., 2019), and this personal music-listening is likely to contribute in varying extents to people’s familiarity with a song and subsequent earworm episodes (e.g., Williamson et al., 2012). A song’s catchiness is therefore somewhat inseparable from exposure, and exposure to the song itself is difficult to measure given the multiple means by which people access music.

This challenge was highlighted in our previous work (Killingly et al., 2021), where it was evident that factors such as the enjoyment of a song and desire to sing along were also associated with previous exposure to the music (in terms of participants’ perceived familiarity). While songs tended to vary in terms of how familiar people were with them, there were consistent significant relationships among participants’ ratings of their enjoyment, familiarity with, and desire to sing along to different songs, and self-reported earworms, indicating that higher levels of familiarity were positively associated with higher levels of enjoyment and the desire to sing along. Even when prior familiarity to the songs was controlled (Exp. 2; Killingly et al., 2021) and matched across conditions insofar as possible, the songs that were rated either high or low in terms of the desire to sing along significantly differed on the ratings of enjoyment and familiarity, such that the songs people wanted to sing along with were also those that they perceived to be more familiar and enjoyable. Hence, for previously heard music, determining the relative contribution of catchiness, the desire to sing along, and perceived familiarity itself represents an intractable problem.

To overcome the issue of prior familiarity, one strategy might be to use musical stimuli which has not been previously heard by the participants, and therefore manipulate exposure to the music within the experiment. This method was successfully employed in an experience sampling study by Byron and Fowles (2015), wherein it was demonstrated that novel music was more likely to be reported as an earworm by participants who heard a song six times compared with those who heard it two times. This finding highlights the importance of repeated exposure in the development of an earworm. Nonetheless, it is worth considering that only two stimulus songs were used for the Byron and Fowles (2015) study, and participants were exposed to either one or the other. It is thus difficult to know the extent to which song-level characteristics may have contributed to the effects produced. Again, with only subjective measures of an earworm experience (even with the benefits of experience sampling methodology), these findings still assume that participants had sufficient awareness of their earworm episodes to accurately self-report them.

In terms of the paradigm employed by Killingly et al. (2021), it remains an open question whether interference in serial recall would be observed following the presentation of previously unheard music, and if not, how much prior exposure to the music is required for it to elicit this interference. Hence, this study investigated whether a novel piece of catchy music can elicit an earworm, as manifested in working memory performance, and whether this effect is influenced by the extent of exposure to the music. Participants were presented with four unfamiliar choruses in one session (with number of presentations manipulated), and their serial recall was tested during and following presentation of each chorus the following day. In line with previous findings (Salamé & Baddeley, 1989), it was expected that concurrent presentation of music would reduce the performance on a serial recall task, and that we would replicate the effect whereby particularly catchy songs trigger continued working memory interference following their presentation (Killingly et al., 2021). Our key prediction for this study was that greater exposure to a song in the first session would engender greater interference following its presentation in the second session.

## Method

### Participants

The sample comprised 44 participants (35 female). Participants were recruited from the community, via social media advertisements, and from the Queensland University of Technology undergraduate psychology cohort. Participants from the community were reimbursed for travel with a gift voucher (US$10 in value), while students were awarded course credits in recognition of their participation. Participants’ ages ranged from 17 to 46 years (*M* = 21.14, *SD* = 5.1).

The sample had initially included an additional 8 participants, with the aim of recruiting enough to ensure a minimum of 12–13 participants hearing each of the four songs at each level of exposure. However, two were excluded who reported high baseline familiarity with the songs. In addition, for six of the participants, it appeared that the digit span test did not accurately estimate their true serial recall capacity, as they achieved less than 60% accuracy when subsequently tested using their estimated digit span. These participants were therefore not included in analyses.

### Materials

#### Songs

Eight candidate earworm songs were sourced from a UK website (theundiscovered.uk) which aims to promote “undiscovered” artists. These songs were subjected to pilot testing with an independent sample (*N* = 10), who rated each on a scale from 0 to 100 on characteristics, such as catchiness, familiarity, enjoyment, and the desire to sing along. The main purpose of pilot testing was to ascertain that these songs were not commonly known. Average ratings of the eight songs are shown below in Table 1, showing that songs were generally enjoyed by pilot participants and were virtually unheard of.

**Table 1.** Average song ratings for pilot study.

	*M*	*SD*
Familiarity (/100)	2.59	9.33
Enjoyment (/100)	42.45	28.15
Desire to sing along (/100)	23.33	22.47
Catchiness (/100)	38.66	27.30

Based on the ratings of 8 obscure songs by 10 participants.

Four songs were selected for use in the main study, and these songs represented a range in terms of pilot participants’ average ratings on the dimensions of catchiness and the desire to sing along, rather than those rated highest on a combination of these dimensions. These were “Healing” by Oh Honey, “Don’t Tell” by Mansions on the Moon, “Believer” by The Paper Lions, and “Running Away” by Royal Foundry. To maximally expose participants to these songs within the short timeframe of the study, and given that earworms are most commonly experienced for choruses (Beaman & Williams, 2010), the chorus of each song was isolated and looped seamlessly for a duration of 2 min. In pilot testing, no song had a mean familiarity rating higher than 8 (although variability among familiarity ratings was high for the song “Believer”; see Table 2).

**Table 2.** Average pilot ratings for songs chosen for experiment.

Song	Familiarity		Enjoyment		Desire to sing		Catchiness	
	*M*	*SD*	*M*	*SD*	*M*	*SD*	*M*	*SD*
Believer	7.80	19.06	38.10	28.56	21.10	18.58	33.20	20.75
Don’t tell	0.10	0.32	31.60	26.85	18.60	23.80	39.10	30.64
Healing	4.00	9.66	48.20	33.75	25.20	25.59	48.20	32.18
Running away	2.00	4.22	46.30	28.81	23.80	21.49	35.70	21.06

Songs were rated out of 100.

#### Digit span task

Short-term memory capacity was measured for each individual participant using a digit span task. For every individual, the task commenced with the simultaneous presentation of three random digits, which appeared for an equivalent number of seconds, and the instruction to remember the digits in the presented order. Once the sequence had disappeared, the participant was asked to type it in, and press “Enter.” If the response was correct, the sequence in the subsequent trial would increase in length by one digit (e.g., correct recall of the three digits would result in a sequence of four random digits); however, incorrect responses were followed by trial in which the length was decremented by one digit (or remained at three digits). Following an incorrect response, two correct successive trials at the reduced sequence length were required before the number of digits increased. Eight reversals were required, and the average of the digits at the most recent six reversals was taken to be the individual’s digit span, resulting in an 80% accuracy threshold.

#### Serial recall task

Participants undertook a number of serial recall trials within short testing blocks. In each trial, a sequence of random digits was presented on the screen, with the digits appearing simultaneously for a duration commensurate with 1 s per digit. Participants were instructed to memorise the digits in the presented order. The number of digits and the number of seconds for which they were presented were determined by the individual’s digit span—that is, a person with a span of six digits would view six-digit sequences for a total duration of 6 s. Once the sequence disappeared, participants were asked to type in the digits and were able to modify current responses using backspace as required. Response time was not limited, with participants being required to press “Enter” to initiate the next trial. Trials were scored as correct if all digits were entered in the order of appearance. There was no interstimulus interval, and trials continued in this manner until the experimenter indicated that the testing block was finished.

### Procedure

Participants attended two experimental sessions which were 1 day apart. The digit span task was undertaken at the outset of the first session, and participants completed subsequent serial recall tasks at their average digit span. A baseline of serial recall performance was obtained, in which participants undertook the task in silence. Following this block, participants rated the four song choruses (presented in random order via Qualtrics^®^; Qualtrics, Provo, UT) in terms of perceived familiarity, enjoyment, desire to sing along, and catchiness. For the remainder of the first session, participants completed 20 blocks of serial recall, alternating between hearing music (in the 10 odd blocks) and silence (during the 10 even blocks). During each of the music blocks, a seamlessly looped version of a song chorus was presented for a duration of 2 min.

In the first session, for every participant, one looped chorus was presented four times over the session, one was presented three times, one was presented twice, and one only once. Exposure to the four song choruses was randomly counterbalanced among participants, such that each of the 2-min-looped choruses was presented at one of the four exposure levels, 11 times across the 44 participants. The order in which the choruses were presented was also randomised within the session.

In the second session, participants undertook a baseline block of serial recall in silence, before any music had been heard. Following baseline, a further eight testing blocks were conducted using the same task. During the odd-numbered blocks (1, 3, 5, and 7), participants were concurrently presented with each of the four looped choruses (in random order). During the even blocks (2, 4, 6, and 8), the serial recall task was completed in silence following the presentation of the music to determine whether the music continued to be actively rehearsed in working memory. In both sessions, duration of each block of serial recall trials was approximately 2 min, and the average number of serial recall trials within each block was 5.26. Participants were then asked to rate each of the song choruses again as they had at the outset. This time, they were also asked whether each song had been experienced as an earworm during the session or between the two sessions.

## Results

### Perception of songs

The novel choruses were highly effective in producing earworms, with 68% of participants self-reporting having experienced at least one as an earworm during the second session, while 52% reported experiencing earworms between the sessions. To investigate whether a recency effect was present in the self-reported earworms, a generalised estimating equation (GEE) was conducted using the song order (four levels) as a predictor and earworm during the silent blocks as the dichotomous outcome variable. Results were significant, χ^2^(3) = 9.94, *p* = .019, and follow-up simple contrasts indicated that the final song was reported significantly more frequently (32% of the time) than the first (24%), second (25%), or third (20%) song presented (*p*s < .05). Hence, participants more frequently tended to report their most recently heard song in Session 2 as an earworm.

Participants’ self-reported familiarity with the choruses was very low at the outset of the study, with mean ratings of each chorus ranging from 1 to 3.7 (out of 100). For initial song ratings, there were no significant differences among any of the choruses in terms of baseline familiarity, desire to sing along, or perceived catchiness. However, there was a small effect of enjoyment, *F* (3, 135) = 5.58, *p* = .001, with follow-up tests indicating that Believer was enjoyed more than the other songs on average (*p*s < .05). Table 3 shows the mean chorus ratings in each session.

**Table 3.** Mean ratings of song choruses in each session.

	Session 1		Session 2	
	*M*	*SD*	*M*	*SD*
Familiarity (/100)	2.86	8.35	37.05	27.94
Enjoyment (/100)	33.69	24.53	32.40	27.29
Desire to sing along (/100)	29.50	27.77	36.78	30.14
Catchiness (/100)	40.28	28.16	43.41	30.68

Songs were rated out of 100.

**Figure 1.**
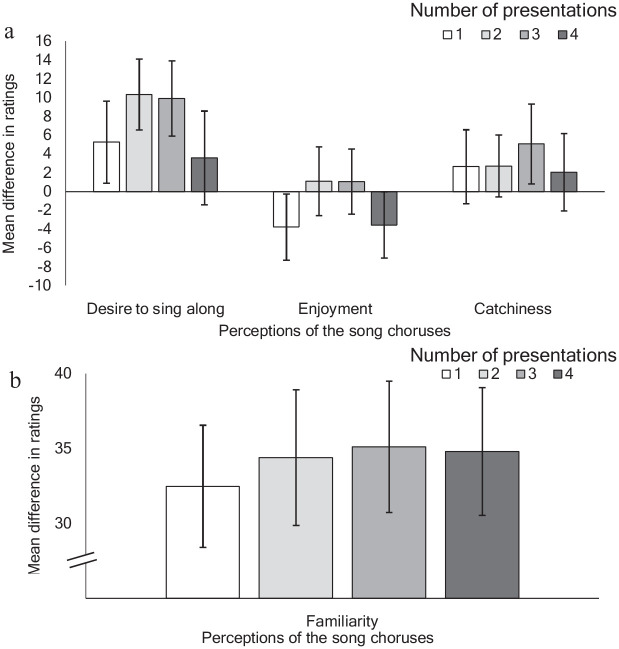
Mean difference in song ratings before and after experiment, according to level of exposure. Panel a: Desire to Sing along, Enjoyment, Catchiness. Panel b: Familiarity. *Note.* Songs were rated on a scale of 0–100 in each session. Error bars represent ± 1 *SE*.

Figure 1 represents changes in the perceptions of the songs before and after the two sessions, split according to the level of exposure in Session 1. Perceived familiarity with the songs appeared to increase linearly with increased exposure, plateauing at the fourth presentation, while desire to sing along, catchiness, and enjoyment were characterised by curvilinear trends. Notably, enjoyment of the songs actually decreased between sessions, for very low exposure (one presentation) or high (four presentations). The error bars (standard error) indicate a large degree of variability in these rating changes.

**Figure 2.**
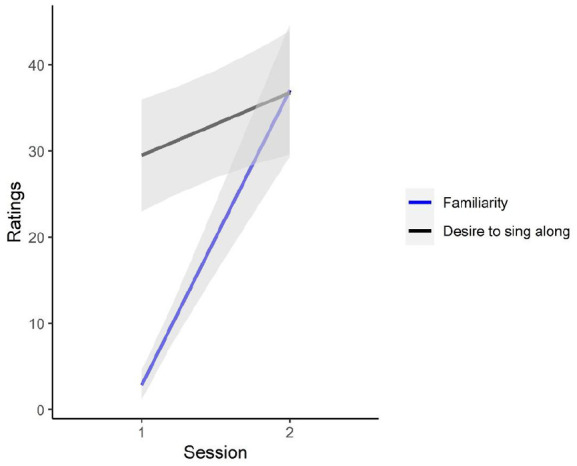
Change in ratings between Sessions 1 and 2. *Note.* Confidence bands represent 95% confidence intervals.

Linear mixed models were employed to assess whether these ratings changed significantly between the sessions, and whether this varied as a result of exposure or according to song. Linear mixed modelling allows for the simultaneous modelling of item- and subject-level attributes without violating assumptions of independence (Baayen et al., 2008). Four analyses were conducted using each of the four ratings as an outcome variable (familiarity, catchiness, enjoyment, and desire to sing). Each factorial model included the three predictor variables of exposure (four levels; number of times each chorus was presented during the first session), song (four levels; each of the choruses), and session (two levels). Random slopes were included for the effect of session, given the likelihood that the change in ratings might vary on an individual basis. Marginal pseudo-*R* effect sizes are reported for each analysis (Nakagawa & Schielzeth, 2013). The models evaluating the ratings of catchiness and enjoyment yielded non-significant results. For ratings of familiarity (model *R*^2^ = .42), there was a main effect of session, such that ratings increased significantly from the first session to the second, *F*(1, 41.9) = 77.93, *p* < .001. No other effects were observed. The desire to sing along also increased significantly from the first to the second session, *F*(1, 39.3) = 6.15, *p* = .018, with no other effects observed (model R^2^ = .05). Figure 2 displays the estimated marginal means for increases in familiarity and the desire to sing along across the experiment.

We also investigated whether self-reported ratings of the songs, as measured in Session 2, were related to self-reported earworms during the session, and whether these relationships were moderated by prior exposure to the songs. Separate GEEs were conducted for each type of rating (familiarity, catchiness, desire to sing along, and enjoyment), each of which employed self-reported earworm frequency (dichotomous) as the outcome variable. Each model included the main effects of song rating, exposure, and the interaction between them. This type of analysis is appropriate for repeated measures data where the outcome variable is dichotomous, as it accounts for responses being correlated within subjects and not normally distributed (Ballinger, 2004). In the model testing familiarity, ratings significantly predicted whether songs were reported as earworms, χ^2^(1) = 9.1, *p* = .003, while exposure and the interaction term did not. Similarly, the model testing the desire to sing along produced a significant effect of ratings, χ^2^(1) = 6.8, *p* = .009, with no other significant effects. For the analysis of enjoyment, no significant effects were observed. Finally, catchiness ratings were significantly related to whether songs were reported as an earworm, χ^2^(1) = 8.5, *p* = .003, with no other effects observed.

### Serial recall accuracy after exposure manipulation (Session 2)

Participants’ digit spans ranged from 5 to 8, with an average of 6.64 (*SD* = 1). Serial recall accuracy was scored dichotomously, according to whether whole digit sequences were produced in the correct order. In the second session, participants’ average accuracy on the serial recall task at baseline was 0.92 (*SD* = 0.27).

An analysis was first conducted to investigate the primary question of whether performance following the presentation of the choruses was significantly reduced in comparison to baseline. A GEE using a factor of Condition (five levels; baseline, each of the four exposure levels) yielded a significant main effect, χ^2^(4) = 11.01, *p* = .026. Simple follow-up contrasts were conducted to investigate each level of exposure in comparison to baseline, and these indicated a significant decrease between baseline and performance following choruses heard four times in the first session, χ^2^(1) = 9.13, *p* = .003. No other significant differences were observed.

We then examined whether different choruses elicited interference during or following presentation, and whether this effect was influenced by the extent of exposure from the first session. A factorial GEE was employed, using the factors of Exposure (four levels; how many times the choruses were presented in the first session), Song (four levels; each of the four choruses), and Time (two levels; during and following presentation).

**Figure 3.**
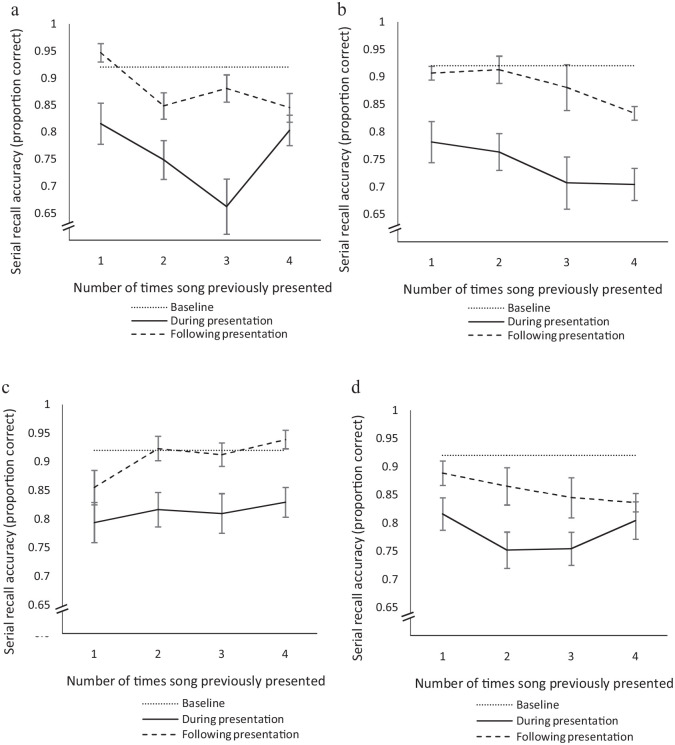
Effect of exposure on serial recall performance before, during, and following presentation of each song. Panel a: Don’t Tell. Panel b: Running Away. Panel c: Believer. Panel d: Healing. *Note.* Error bars indicate ± 1 *SE*.

The analysis yielded significant main effects of Song, Exposure, and Time (see Table 4), which, respectively, indicated that songs varied in the impacts they had on performance, performance was increasingly affected as the number of song presentations increased, and performance was poorer during presentation of music, compared to following. However, there were also significant two-way interactions for Song × Exposure and Song × Time, suggesting that the four songs chosen for the study served to moderate the other two effects. Notably, the absence of a Time × Exposure interaction (or three-way interaction) indicates that the effect of exposure was similar both during and following presentation of the music (see Figure 3).

**Table 4.** Three-way GEE analysis (Exposure × Song × Time) on serial recall performance.

	χ^2^	*df*	*p*
Exposure	9.45	3	.024*
Song	13.30	3	.004*
Time	205.12	1	<.001*
Song × exposure	31.69	9	<.001*
Time × exposure	7.49	3	.058
Song × time	8.65	3	.034*
Song × time × exposure	12.21	9	.202

*Note*. * denotes significance at *p* < .05 level.

The significant Song × Time interaction was followed up by determining the simple effect of Song at each level of Time. Songs significantly varied in their influence on serial recall performance, both during, χ^2^(1) = 12.32, *p* = .006, and following presentation, χ^2^(1) = 11.36, *p* = .010. Notably, it appears that the song Healing generated the most interference following its presentation, potentially reflecting a particularly sticky tune. Bonferroni-adjusted pairwise comparisons indicated that performance significantly differed during presentation of Running Away compared to Believer (*p* = .003), and performance following presentation differed after participants heard Healing compared to Believer (*p* = .005); no other comparisons reached significance.

To follow up the Exposure × Song interaction, simple effects of Exposure at each level of Song were assessed, revealing a significant effect for the songs Don’t Tell and Running Away, *p*s < .005. Polynomial contrasts were conducted for the effect of Exposure for each significant Song chorus. There was a negative linear trend for the song Don’t Tell, χ^2^(1) = 4.7, *p* = .030, such that performance during and following presentation of the song diminished as the number of times previously heard increased. There was also a negative trend for the song Running Away, χ^2^(1) = 11.94, *p* < .001. Simple effects for Healing and Believer were non-significant. Song-level effects, even for novel music, are somewhat unsurprising given the idiosyncratic nature of earworm experiences. However, it is worth noting that the necessity of investigating exposure individually for each song has resulted in smaller cell sizes per level of exposure, and therefore, these trends should be interpreted with caution. Further research is warranted to investigate this effect with a larger sample of participants and different songs.

Figure 3 displays serial recall performance during and following presentation of the songs as a function of prior exposure to the music. Baseline performance (in silence, before presentation of songs) is included as a reference.

## Discussion

The results of this study indicate that prior exposure to a song moderates the extent to which it gets stuck in the head, as indexed by reduced performance on a working memory task. Specifically, repeated exposure to novel music in one session disrupted serial recall performance both during and following presentation of the music a day later. However, this effect appeared to be song-specific, with not all stimuli interfering with working memory in the same way. Overall, serial recall performance following the songs which were presented most frequently (four times) was significantly reduced in comparison to baseline. This study supports previous research which indicates the importance of exposure to music over time in the development of earworms (Byron & Fowles, 2015; Liikkanen, 2012), and it provides further evidence of the effective use of an experimental working memory paradigm to measure earworm experiences (Killingly et al., 2021).

Intriguingly, this study demonstrated that both self-reported familiarity and the desire to sing along with the songs increased significantly between the two sessions, providing something of a corollary to the notion that an earworm episode is characterised by inner mental singing along (Killingly et al., 2021). Ratings of enjoyment did not significantly increase in contrast to research regarding the effects of exposure on liking (Peretz et al., 1998; Szpunar et al., 2004). Typically, a “mere exposure” effect has been demonstrated, whereby repeated exposure to a given stimulus increases preference (Zajonc, 1968). Topolinski and Strack (2009) propose that this effect is mediated by motor simulation of the presented stimuli. In their account, repeated exposure induces automatic sensorimotor simulation, thereby increasing fluency, and it is this increase in fluency which drives the increase in one’s preference towards the stimulus. In this study, the significant increase in the desire to sing along provides a corollary to the notion that the interference is produced by automatic subvocal singing. As people become more exposed to music, they engage in more subvocal rehearsal of the song, increasing their perceptual fluency with the stimuli, and their desire to sing along. It may be this subvocal singing which acts to trigger an earworm, which is then maintained in working memory.

Although the phonological loop has primarily been considered in terms of its ability to assist with complex cognitive tasks, it has been argued that this use may represent an artefact of its more fundamental role in language acquisition (Baddeley et al., 1998). Specifically, it may function chiefly as a mechanism by which novel phonological representations are stored while long-term memories are being made or reinforced. A more recent account of long-term language learning has shown that verbal sequence learning, which underpins language acquisition, is dependent on articulatory motor planning (Sjöblom & Hughes, 2020). We suggest that in the case of hearing words set to music, rather than simply disrupting the rehearsal process, it may be that working memory resources are co-opted by subvocal singing of the song, driven by an instinct to learn or reconsolidate the information. This interpretation of the data suggests that it is the subvocal articulation of an earworm which interferes with serial recall performance, akin to an effect of articulatory suppression, and accords with previous findings showing that interference to articulatory planning reduces self-reported earworms (e.g., Beaman et al., 2015). Alternatively, it may be that the interference to serial recall arises from the auditory imagery generated by that rehearsal, a possibility which remains an open question for future research.

In general, the literature shows that information is often recalled more successfully when learned as part of a song (Calvert & Tart, 1993; Good et al., 2015; Ludke et al., 2014; Schön et al., 2008; Wallace, 1994). Studies show that singing new vocabulary aids in learning a new language (Good et al., 2015; Ludke et al., 2014), and simply listening to text set to melody can enhance one’s recall of the words, as epitomised by the effective use of advertising “jingles” (Alexomanolaki et al., 2007; Yalch, 1991). Research in this area tends to find that the learning advantage derived from pairing information with music is dependent on familiarity with a melody (Tamminen et al., 2017; Wallace, 1994). The present findings suggest a possible mechanism underpinning this learning phenomenon. With increased exposure, novel songs generated greater interference on a task requiring subvocal articulation, potentially as working memory processes were automatically recruited to reinforce newly consolidated long-term memories. It would be interesting to examine whether the effects observed in this study last beyond 24 hr, and whether increasing exposure during the first session beyond four presentations would engender greater interference.

The link between earworms and memory consolidation may be even more generalised than in the case of better recall for information set to music. Recent research has investigated the mediating role that an earworm may serve in the context of music-evoked autobiographical memories. In several experiments in which previously unknown music was paired with short videos, Kubit and Janata (2022) found that participants who subsequently experienced the musical excerpts as earworms were able to recall more details from the videos at a later time, suggesting that the very experience of an earworm can help to consolidate associated event-based memories.

The fact that working memory was significantly disrupted, both during and following presentation, on the basis of previous exposure suggests that objective familiarity with a song has a significant influence not only on the appearance of earworms but also on working memory performance in the presence of background music more generally. Hence, this finding extends on previous research regarding the distracting effects of concurrent vocal music presentation on working memory performance (Alley & Greene, 2008; Perham & Sykora, 2012; Salamé & Baddeley, 1989), indicating that prior exposure to a song moderates this effect, regardless of perceived enjoyment. In addition, and in line with our previous work (Killingly et al., 2021), this study shows that irrelevant background music can continue to produce interference to serial recall beyond its presentation, a finding not observed in previous research investigating the effect of irrelevant sound using non-music stimuli (Macken et al., 1999).

A key strength of this study was the use of novel musical stimuli, with familiarity induced incrementally by repeated presentations. In contrast, previous research in this has relied on participants’ subjective ratings of familiarity (Alley & Greene, 2008; Perham & Sykora, 2012), potentially obscuring the effects of actual prior exposure to a song. Even in this study, despite the clear effects of actual song exposure on working memory, differences in ratings of familiarity between sessions did not vary according to the number of song presentations, suggesting a potential dissociation between subjective sense of familiarity with a stimulus and actual familiarity.

An important avenue for future research would be to investigate more comprehensively the musical characteristics that tend to elicit or strengthen the effects observed. In this study, repeated exposure to two of the four song choruses tended to result in diminished performance following their presentation; in contrast, one of the songs produced less interference the more times it had been heard, albeit this effect was non-significant. At this stage, given the exploratory nature of this study, the small number of songs used, and the relatively small sample size, it would be precipitate to draw any general conclusions regarding musical features of the stimuli used.

Moreover, as this experiment used music with words, it becomes impossible to elucidate the relative contribution of the music and the lyrics in producing the effect. It is also worth noting that in the blocks where music was presented to participants in either session, each of the song’s choruses were repeated for a period of 2 min, and this means that the number of repetitions of a chorus within that segment was not equivalent across different songs. Future research employing this paradigm should ensure that exposure to the different musical stimuli is controlled.

However, these findings represent a promising beginning and may provide an experimental method for systematically investigating the musical features of earworms, while accounting for familiarity with the music. Hence, this study adds to the body of knowledge regarding how exposure to music over time can influence the way in which it is subsequently processed at a cognitive level, and how it contributes to the development of an earworm. Furthermore, these findings have implications for the understanding of working memory and its associated effects, such as that of irrelevant background sound. This study additionally provides some preliminary insights into how long-term memories may be acquired and reinforced. This paradigm could be used to great effect in educational and marketing research; by varying stimuli and exposure levels, it may be possible to determine the effective strategies for the use of music as a learning tool.
